# Effect of ferric carboxymaltose on serum phosphate and C-terminal FGF23 levels in non-dialysis chronic kidney disease patients: post-hoc analysis of a prospective study

**DOI:** 10.1186/1471-2369-14-167

**Published:** 2013-07-31

**Authors:** Merche Prats, Ramon Font, Carmen García, Carmen Cabré, Manel Jariod, Alberto Martinez Vea

**Affiliations:** 1 Nephrology Service.Hospital Universitari de Tarragona Joan XXIII, Universitat Rovira i Virgili, Tarragona, Spain; 2 Unidad de Sistemas de Información de la Gestión, Hospital Universitari de Tarragona Joan XXIII, Tarragona, Spain; 3 Servicio de Nefrología, Hospital Universitari de Tarragona Joan XXIII, Carrer del Doctor Mallafré Guasch 4, 43007, Tarragona, Spain

**Keywords:** Chronic kidney disease, Ferric carboxymaltose, Fibroblast growth factor 23, Hypophosphatemia, Iron deficiency anaemia

## Abstract

**Background:**

Some parenteral iron therapies have been found to be associated with hypophosphatemia. The mechanism of the decrease in serum phosphate is unknown. The aim of this study is to examine the effect of IV ferric carboxymaltose(FCM) on phosphate metabolism and FGF23 levels in patients with chronic kidney disease(CKD).

**Methods:**

This is a post-hoc analysis of a prospective study carried out in 47 non-dialysis CKD patients with iron-deficiency anaemia who received a single 1000 mg injection of FCM. Markers of mineral metabolism (calcium, phosphate, 1,25-dihydroxyvitamin D, PTH and FGF23[c-terminal]) were measured prior to FCM administration and at week 3 and week 12 after FCM administration. Based on the measured levels of serum phosphate at week 3, patiens were classified as hypophosphatemic or non-hypophosphatemic.

**Results:**

Serum phosphate levels decreased significantly three weeks after FCM administration and remained at lower levels at week 12 (4.24 ± 0.84 vs 3.69 ± 1.10 vs 3.83 ± 0.68 mg/dL, respectively, p < 0.0001. Serum calcium, PTH and 1,25-dihydroxyvitamin D did not change over the course of the study. Serum FGF23 decreased significantly from 442(44.9-4079.2) at baseline to 340(68.5-2603.3) at week 3 and 191.6(51.3-2465.9) RU/mL at week 12, p < 0.0001. Twelve patients were non-hypophosphatemic and 35 hypophosphatemic. FGF23 levels decreased in both groups, whereas no changes were documented in any of the other mineral parameters.

**Conclusions:**

In non-dialysis CKD patients, FCM induces reduction in serum phosphate levels that persists for three months. FCM causes a significant decrease in FGF23 levels without changes to other bone metabolism parameters.

## Background

Iron deficiency is common in non-dialysis chronic kidney disease (CKD) patients and is most pronounced in haemodialysis patients [1,2]. Supplementation with oral or intravenous (IV) iron is a common practice in this population. Oral iron therapy is limited by poor gastrointestinal absorption, frequent adverse events and low adherence to treatment. Therefore, IV iron is the preferred method of iron replacement in these patients [3]. Nevertheless, high iron doses can lead to serious adverse consequences such as exacerbation of oxidative stress, inflammation, endothelial dysfunction, immune deficiency and increased tissue iron stores [4,5]. There are also other concerns about currently available IV iron agents, including the potential for immunogenic reaction, dose limitations and the requirement of a test dose, and the desired rate of repletion [6-8]. To overcome these limitations, new IV iron preparations have been introduced, offering higher single-dose options with an acceptable side-effect profile [7].

Ferric carboxymaltose (FCM) is an innovative non-dextran iron complex that is composed of a ferric hydroxide core stabilized by a carbohydrate shell, carboxymaltose, allowing controlled delivery of iron to the target tissues [9]. Unlike earlier forms of IV iron, FCM can be given in a dose providing up to 1000 mg of iron administered as a rapid infusion over 15 min. without the need for a test dose [9]. In predialysis CKD patients and in patients undergoing haemodialysis, FCM is effective and well tolerated, and is associated with few adverse events [8,10,11]. A common adverse event associated withg FCM is a transient, asymptomatic hypophosphatemia, which has been primarily reported in patients with postpartum iron-deficiency anaemia and in patients with iron-deficiency anaemia due to heavy uterine bleeding who were treated with large doses of FCM [12,13]. However, hypophosphatemia is neither widely acknowledged nor documented in CKD patients. In fact, transient hypophosphatemia has only been reported in 2.7% of non-dialysis CKD patients [9] and in 4.3% of a CKD population [14] treated with FCM, but was not mentioned in the study conducted by Covic A et al. [8] on anaemic haemodialysis patients or in the study by Grimmelt A et al. [10] on predialysis CKD patients treated with variable doses of FCM.

The cause of hypophosphatemia during IV iron therapy remains unclear. It has been observed after the administration of other IV iron preparations such as iron saccharide [15] or iron polymaltose [16], but not with low molecular weight iron dextran [7], ferric gluconate [17] or iron isomaltoside [18]. Because hypophosphatemia has been reported in association with stimulated erythropoiesis in other haematopoietic disorders [19-21], it has been suggested that iron-induced hypophosphatemia might be the result of an increased cellular uptake of phosphate during erythropoiesis [13]. However, the main mechanism of iron-induced hypophosphatemia seems to be renal phosphate wasting [22]. Impaired tubular phosphate reabsorption has been reported during treatment with saccharated ferric oxide [15] and with iron polymaltose [16]. In addition to renal phosphate loss, an inhibition of renal 25 (OH) D 1α-hydroxylase activity and decreased 1,25-dihydroxyvitamin D levels were also reported in these cases [15,16].

Since parenteral iron may have a direct toxic effect on proximal renal tubular cells, renal phosphate loss could be the consequence of proximal tubular dysfunction induced by iron therapy [23]. Nevertheless, the dual inhibition of tubular phosphate reabsorption and 1α-hydroxylation of vitamin D observed during iron therapy, suggests that a phosphatonin hormone, fibroblast growth factor 23 (FGF23), may play a role in the hypophosphatemia induced by IV iron. In fact, hypophosphatemia with impaired tubular reabsorption, low 1,25(OH)_2_ vitamin D and high FGF23 levels has been reported in anaemic patients with normal renal function treated with saccharated ferric oxide or iron polymaltose [15,16], supporting an etiologic role of FGF23 in the development of hypophosphatemia. In haemodialysis patients, IV low molecular weight iron dextran [24] or saccharate ferric oxide [25] also induce an increase in FGF23 levels and a decrease in PTH levels but no changes in serum phosphate.

To investigate the possible link between FCM therapy and phosphate metabolism, and to ascertain the possible mechanisms of FCM-associated hypophosphatemia, a post-hoc analysis was conducted on existing data from our previous study evaluating FCM therapy in a group of predialysis CKD patients with iron-deficiency anaemia [26].

## Methods

### Patients

This study was performed as a post-hoc analysis of a prospective study carried out in non-dialysis CKD patients treated with FCM [26]. Subjects ≥ 18 years of age with an estimated glomerular filtration rate < 60 ml/min/1.73 m^2^, serum haemoglobin < 11 g/dL and either serum ferritin < 100 ng/dL or transferrin saturation < 20%, were included in the study. Exclusion criteria included oral or IV iron treatment or blood transfusion during the three-month period prior to the study, active infection, active bleeding, anticipated dialysis in the next three months, and known hypersensitivity to other IV iron preparations.

The study was approved by the ethics committee of the Joan XXIII University Hospital of Tarragona, and all participants provided written informed consent.

### Study design

All patients received a single IV infusion of FCM (Ferinject^R^, Vifor Pharma, Switzerlan) over 30 min. at a maximum dose of 1000 mg(15 mg/Kg) dilute in 250 mL normal saline, and were monitored for 12 weeks. During the study period, dietary phosphate intake and doses of erythropoiesis stimulating agents, active vitamin D, phosphate binders and cinacalcet hydrochloride were kept unchanged.

According to levels of serum phosphate obtained at week three, patients were classified as either hypophosphatemic or non-hypophosphatemic if phosphate levels decreased or remained unchanged or even increased compared to baseline levels.

### Biochemical determinations

Blood samples were taken at baseline and at the end of week 3 and week 12 of the follow-up period. Serum haemoglobin, transferrin saturation, ferritin, calcium, phosphate and creatinine were determined by means of standard laboratory methods. Serum parathyroid hormone was determined by means of chemiluminescence(DPC Laboratories, Miami, FL, USA). Baseline levels of 25-hydroxy vitamin D were measured by chemiluminiscence(Diasorin, Italy). Serum 1,25 (OH)_2_ D levels were measured by RIA(Diasource Immuno Assays, Belgium). Serum FGF23 concentrations were measured with the human FGF23 (C-terminal) ELISA kit (Immutopic, Inc, San Clemente, CA, USA). This C-terminal assay use two antibodies directed against the carboxyl-terminal portion of the molecule and detects both total intact and C-terminal FGF23 [27]. The estimated glomerular filtration rate(GFR) was calculated using the abbreviated MDRD formula [28].

### Statistical analysis

Statistical analysis was performed using the SPSS/PC, v.15 statistical package (IMC, Chicago, IL). Values are expressed as mean ± SD, and in case of PTH and FGF23 levels as median(range). Because serum FGF23 levels were not normally distributed, log-transformed values were used in the analysis. Mean levels were compared between time points using the repeated measurements ANOVA. The univariate relationship between variables was assessed by means of Spearman correlations. A p value of < 0.05 was considered statistically significant.

## Results

### Study population

A total of 50 patients were enrolled in the study. Two subjects were withdrawn during the study because of bleeding, and one patient was excluded due to non-adherence to study protocol. Thus, 47 patients were included in the final analysis. The baseline demographics and clinical characteristics are shown in Table 1. Mean age was 72 years, and the most common primary renal disease was vascular nephropathy. Mean serum creatinine and estimated GFR were 2.5(0.9) mg/dL and 26.1(0.4) ml/min/1.73 m^2^, respectively. Most patients(85%) were in CKD stages 3–4. Only four patients were treated with phosphate binders, whereas 18 patients were receiving active vitamin D: paricalcitol(16 patients), calcitriol(2 patients). The mean cumulative dose of FCM was 971.7(69.3) mg. and there were no adverse events associated with FCM infusion during the study period.

**Table 1:** Baseline demographic and clinical characteristics

**Characteristics**	**Value**
Age(years)	72.2(11.6)
Male/Female	23/25
Primary renal disease	
Vascular nephropathy	23(49%)
Glomerulonephritis	2(4%)
Interstitial nephropathy/polycystic kidney disease	3(6%)
Diabetes	12(26%)
Unknown	7(15%)
Chronic kidney disease stage	
Stage 3	13(28%
Stage 4	27(57%)
Stage 5	7(15%)
Use of erythropoiesis stimulating agents	23(49%)
Use of phosphate binders	4(8.5%)
Use of active vitamin D	18(38%)
Use of cinacalcet hydrochloride	1(2%)
Serum creatine, mg/dL	2.56(0.95)
eGFR, ml/min per 1.73 m^2^	26.1(10.4)
Haemoglobin, g/dL	10(0.7)
Ferritin, ng/mL	67.8(61.7)
Transferrin saturation, %	14.6(6.4)
Calcium, mg/dL	9.3(0.4)
Phosphate, mg/dL	4.2(0.8)
PTH, pg/mL	138.1(2.5-600)
25-hydroxyvitamin D, ng/ml	12.8(6.6)
1,25 dihydroxyvitamin D, pg/mL	9.7(4.4)
Fibroblast growth factor 23, RU/mL	442(44.9-4079.2)

### Effect of FCM on iron-deficiency anaemia

Changes in iron indices and haemoglobin concentration before and after FCM are presented in Table 2. Haemoglobin levels and serum ferritin and transferrin saturation rose significantly and remained elevated at the end of the study.

**Table 2:** Effect of FCM on haemoglobin concentration, iron indices and mineral metabolism parameters

	**Baseline**	**Week 3**	**Week 12**	**p-value**
Haemoglobin, g/dL	10(0.7)	10.8(0.8)	11.4(1.3)	<0.0001
TSAT, %	14.6(6.4)	28.9(10)	25.6(12.7)	<0.0001
Ferritin, ng/mL	67.8(61.7)	502.5(263.3)	230(144.6)	<0.0001
Calcium, mg/dl	9.3(0.4)	9.3(0.5)	9.3(0.5)	ns
Phosphate, mg/dL	4.2(0.8)	3.6(1.1)	3.8(0.6)	<0.0001
PTH, pg/ml	138.1(2.5-600)	124(2.5-736)	106.5(2.5-613)	ns
1,25(OH)_2_ D, pg/mL	9.7(4.4)	10(3.7)	10.4(5.4)	ns
FGF23, RU/mL	442(44.9-4079.2)	340(68.5-2603.3)	191.6(51.3-2465.9)	<0.0001

*TAST*: transferrin saturation.

### Effect of FCM on mineral parameters

The biochemical parameters of mineral metabolism at baseline and after FCM infusion are shown in Table 2. Serum phosphate levels decreased significantly three 3 weeks after the FCM administration, and remained at lower levels at week 12. Serum calcium, PTH and 1,25-vit D levels did not change during the study. However, serum FGF23 decreased significantly from 442(44.9-4079.2) RU/mL at baseline to 191.6(51.3-2465.9) RU/mL at the end of the study. Changes in serum phosphate and C-terminal FGF23 were similar in all stages of CKD. There were no significant changes in estimated GFR during the study.

The relationship between serum phosphate levels and mineral metabolis parameters are shown in Table 3. A significant association was found between serum phosphate and FGF23 levels at week three. Serum phosphate was negatively correlated with estimated GFR throughout the study. There was no correlation between the decline in phosphate and FGF23 levels at week 3 or at week 12.

**Table 3:** Relationship between phosphate levels and other mineral metabolism parameters and estimated GFR

	**Baseline**		**Week 3**		**Week 12**	
	**Phosphate**		**Phosphate**		**Phosphate**	
	r	p-value	r	p-value	r	p-value
**Baseline**						
Calcium	0.04	ns				
PTH	0.16	ns				
FGF23	0.28	0.05				
1,25(OH)_2_ D	−0.29	0.05				
eGFR	−0.62	<0.0001				
**Week 3**						
Calcium			0.01	ns		
PTH			0.01	ns		
FGF23			0.47	0.001		
1,25(OH)_2_ D			0.20	ns		
eGFR			−0.53	<0.0001		
**Week 12**						
Calcium					−0.02	ns
PTH					−0.21	ns
FGF23					0.19	ns
1,25(OH)_2_ D					−0.16	ns
eGFR					−0.39	0.007

Baseline serum FGF23 levels significantly correlated with phosphate(r = 0.28, p = 0.05), transferrin saturation (r = −0.40, p = 0.001), ferritin(r = −0.30, p = 0.007) and estimated GFR(r = −0.48, p = 0.002). No correlation between FGF23 and calcium, 1,25-vit D or PTH was found.

### Hypophosphatemic vs non-hypophosphatemic patients

Twelve patiens were non-hypophosphatemic and 35 hypophosphatemic. Age, use of active vitamin D or phosphate binders, dose of infused FCM, baseline estimated GFR, haemoglobin concentration, iron index, and serum calcium, phosphate, PTH, 25-hydroxy vitamin D and 1.25 vit D levels were similar in both groups (Table 4). Nevertheless, there was a trend toward higher baseline FGF23 levels in non-hypophosphatemic than in hypophosphatemic patients, but it did not reach statistical significance. No correlation was demonstrated between the percentage reduction of serum FGF23 and that of serum phosphate at any time in either of these groups.

**Table 4:** Baseline characteristics of hypophosphatemic and non-hypophosphatemic patients

	**Hypophosphatemic**	**Non-hypophosphatemic**	**p-value**
	**(n = 35)**	**(n = 12)**	
Age(years)	72.3(11.2)	72.5(13.5)	ns
Serum creatinine, mg/dL	2.5(1)	2.7(0.7)	ns
eGFR, ml/min/1.73 m^2^	26.6(10.4)	24.6(10.4)	ns
Haemoglobin, g/dL	10(0.6)	10(0.8)	ns
Ferritin, ng/mL	64.1(58.3)	78.5(72.2)	ns
Transferrin saturation, %	14.6(6.4)	14.6(6.7)	ns
FCM, dosage(mg)	966.4(77.9)	987(31.6)	ns
Use of phosphate binders, n(%)	3(8.5)	1(8.3)	ns
Use of active vitamin D, n(%)	14(40)	4(33.3)	ns
Calcium, mg/dL	9.3(0.4)	9.3(0.6)	ns
Phosphate, mg/dL	4.1(0.8)	4.5(0.7)	ns
PTH, pg/mL	147.8(31.6-600)	84(2.5-404.9)	ns
25-hydroxyvitamin D, ng/mL	12.5(6)	13.8(8.5)	ns
1,25 dihydroxyvitamin D, pg/mL	10.3(4)	8.55(2)	ns
Fibroblast growth factor 23, RU/mL	437.5(44.9-2415)	844.1(148.4-4079.2)	ns

### Effect of vitamin D therapy on mineral metabolism parameters during FCM treatment

Eighteen patients were receiving vitamin D therapy at the time of the study and 29 were not. Baseline mineral metabolism parameters and changes in calcium, phosphate, PTH and 1,25- vit D throughout the study did not differ between untreated and treated patients. However, the observed decrease in FGF23 levels in the group of patients treated with vitamin D was lower than that observed in the untreated patients: -6.8% (−84.7% to 325.6%) and −36.1% (−91.6% to 52.4%) at week 3, respectively, and −21.7% (−66.5% to 46%) and −55.7% (−92.3% to 90.3%) at week 12, respectively, p = 0.03.

## Discussion

In this post-hoc analysis we showed that a single infusion of FCM in non-dialysis CKD patients causes significant and prolonged reduction in serum phosphate levels and a decrease in C-terminal FGF23 levels, but no changes in serum calcium, PTH or 1,25-vit D levels.

### FCM and hypophosphatemia

Transient mild-moderate asymptomatic hypophosphatemia is common among patients receiving FCM, especially in patients with postpartum iron deficiency anaemia or uterine bleeding [12,13]. Hypophosphatemia has also been documented with other available IV iron preparations such as iron saccharide complexes [15] or iron polymaltose [16], but not with low molecular weight iron dextran [7] or iron isomaltoside [18]. The mechanisms of the decrease in serum phosphate are unknown, and the differences in the hypophosphatemic effect observed with various IV iron preparations remain unclear.

Several mechanisms have been suggested to explain the hypophosphatemia induced by IV iron therapy, including the restoration of normal erythropoietic activity with cellular uptake of extracellular phosphate [13], phosphate binding to FCM via electrostatic interaction [29], and renal phosphate wasting due to the direct toxic effect of iron on renal tubular cells [22,23]. Finally, it has been suggested that FGF23, by reducing renal phosphate reabsorption and inhibiting 1α-hydroxylation of vitamin D, may play an etiologic role in the development of iron-related hypophosphatemia [15,16]. In our study, the persistence of hypophosphatemia over 12 weeks despite the discontinuation of FCM argues against the extracellular uptake of phosphate or phosphate binding to FCM, as mechanisms of hypophosphatemia. We did not measure the fractional tubular reabsorption of phosphate, which may have allowed us to confirm the effect of FCM on renal phosphate transport.

It has been proposed that IV iron leads to hypophosphatemia by direct proximal tubular damage, resulting in a specific decrease in tubular phosphate reabsorption, but without other manifestations of a generalized proximal tubular toxicity, such as glucosuria or aminoaciduria [16]. The discontinuation of iron usually results in the rapid normalization of this disorder [25], suggesting that other mechanisms, rather than direct renal tubular damage, may be involved in the onset of hypophosphatemia. Furthemore, the high molecular mass of some IV iron preparations such as iron polymaltose (462,000 daltons) or FCM (150,000 daltons) may make their excretion through urine difficult, arguing against the direct toxic effect of iron on renal tubular cells [30]. However, it is possible that tubular damage induced by iron therapy is more likely in subjects with pre-existing CKD and anaemia treated with high doses of iron preparations. In fact iron accumulates in proximal tubular lysosomes in patients with proteinuria and/or chronic renal failure [31]. In rats subjected to a partial nephrectomy, the accumulation of iron in the cells of the proximal tubule was found to correlate with proteinuria, tubule damage and impairment of glomerular filtration [32]. Recently Tolouian et al. reported extensive deposits of iron in the mesangium and in the interstitial histiocytes in a patient with advanced diabetic nephropathy treated with ferumoxytol, another high molecular weight iron preparation, whereas renal iron deposition was not observed in another patient receiving IV iron dextran [33]. It seems that the intrinsic cytotoxic potential of IV iron may be dependend on the nature of the carbohydrate polymers employed, which appear to determine cellular iron uptake and the capacity of these compounds to induce cellular damage [34].

### FCM and FGF23

In our study, FCM induces a decrease in FGF23 levels. This finding differs from previous studies which described an increased in intact FGF23 concentrations after IV iron therapy in patients with iron deficiency anaemia, normal renal function and normal baseline FGF23 levels [15,16,30]. In haemodialysis patients, IV saccharated ferric oxide or low molecular weight iron dextran resulted in an elevation in intact FGF23 levels accompanied by a reduction in PTH levels, but without changes in phosphate levels [24,25]. However, in a recent study in a dialysis population, a negative relationship was found between iron administration and serum intact FGF23 levels [35].

Our data suggest that in the CKD population, FCM has a negative effects on C-terminal FGF23 levels. Since FGF23 levels are highly regulated by serum phosphate or metabolic changes associated with hypophosphatemia [36], we can postulate that the resulting fall in phosphate levels as a consequence of iron-tubular toxicity would be expected to suppress FGF23 levels. However, in our study we did not find a relationship between the decline in phosphate and FGF23 levels, and changes in FGF23 levels did not differ between hypophosphatemic and non-hypophosphatemic patients, suggesting that the decrease in FGF23 was not related to the reduction in serum phosphate.

An unexpected finding in our study was the fact that FCM had no effect on 1,25-vit D or PTH levels. In subjects with normal renal function, FCM induces increased urinary fractional excretion of phosphate, decreased 1,25- vit D levels and increased PTH levels [37]. On the other hand, in hemodialysis patients, some iron preparations induce a reduction in PTH levels and no changes in 1,25- vit D levels [24,25]. It may be speculated that in CKD patients, the effect of intact FGF23 to suppress renal production of 1,25 vit D may be less pronounced, and the direct effect of FGF23 to suppress PTH secretion might be augmented.

Lower serum iron concentrations are associated with elevated FGF23 levels measured with a C-terminal FGF23 but not with an intact FGF23 assay [38,39]. It has been suggested that iron deficiency may result in an increased rate of proteolytic cleavage of the intact and biologically active hormone to inactive C- and N- terminal fragments [40,41], although a recent western blotting analysis did not confirm this hypothesis [42].

In a population with iron deficiency, improvement in iron status with iron sulphate was associated with a decrease in plasma C-terminal FGF23 levels [43]. Thus, we suggest that FCM may have a negative effect on the production and/or secretion of FGF23 by bone cells. Iron overload is associated with osteoporosis, with a decrease in bone formation without changes in bone resorption [44]. Iron could slow down bone formation by inhibiting osteoblast progenitor cell differentiation [45]. FGF23 is expressed in osteoblast/osteocyte lineage cells [38], and osteoblastic bone formation is a potent modulator of FGF23 production and release into the bloodstream [46]. Therefore, we can postulate that if iron therapy has a negative effect on bone formation, it may also have a blunting effect on C-terminal FGF23 synthesis and secretion from osteoblasts. Finally, some iron preparations, such as FCM, may simultaneously inhibit FGF23 degradation in osteocytes, leading to an increase in intact FGF23 and reduced serum phosphate levels [37].

### FCM and vitamin D treatment

In our study we also explored the influence of active vitamin D treatment on changes in FGF23 levels during FCM infusion. In the group of untreated vitamin D patients, FGF23 levels decreased, while this response was mitigated in the group of patients given vitamin D. These results are in keeping with the observation that vitamin D stimulates FGF23 production in osteoblasts [47], and also increases serum FGF23 levels in haemodialysis patients [48]. Therefore, we suggest that treatment with active vitamin D may play a role for maintaining elevated levels of FGF23 in non-dialysis CKD patients receiving FCM.

### Limitations of the study

The strengths of this study include the use of a prospective sample of non-dialysis CKD patients treated with FCM with a long follow-up period, and uniform bone mineral metabolism data acquisition. There are, however, several potential limitations that deserve mention. Firstly, this was a secondary analysis of an open-label single-arm clinical trial that lacked a concurrent control group. These limitations prevented definitive conclusions to be drawn about the effect of FCM on serum phosphate in CKD patients. Secondly, we did not measure the fractional tubular reabsorption of phosphate and bone turnover markers were not available in our cohort. Thus, the direct effect of FCM on renal phosphate transport and bone metabolism and FGF23 synthesis could not be assessed. Thirdly, we did not determine the biologically active intact FGF23. This is important because of the known divergence of intact FGF23 and C-terminal FGF23 levels in the setting being studied, namely iron deficiency. Finally, the small sample size limited the ability of the study to analyse all of the factors that may have influenced the changes in phosphate levels.

## Conclusions

Treatment with FCM for non-dialysis CKD patients with iron-deficiency anaemia induces reduction in serum phosphate levels that persists for three months. FCM causes a significant decrease in FGF23 levels without changes in other bone metabolism parameters. Further studies are needed to fully bring to light the mechanisms by which FCM modulates phosphate levels and disrupts FGF23 metabolism in CKD patients, and to determine whether long-term administration of FCM may induce hypophosphatemic osteomalacia in this population.

## Competing interest

The authors declared that they have no competing interest.

## Authors’ contributions

MP: Conception and design; Data analysis and interpretation; Provision of study patients; Manuscript writing; Final approval of manuscript. RF: Provision of study patients, Final approval of manuscript. CG: Provision of study patients; Final approval of manuscript, MJ: Data analysis and interpretation; Final approval of manuscript, AMV: Conception and design; Data analysis and interpretation; Provision of study patients; Manuscript writing; Final approval of manuscript. All authors read and approved the final manuscript.

## Pre-publication history

The pre-publication history for this paper can be accessed here:

http://www.biomedcentral.com/1471-2369/14/167/prepub
